# Chloroform in Hooping-Cough

**Published:** 1854-01

**Authors:** 


*Chloroform in Hooping-Cough.*—Dr. Churchill recommends chloroform in hooping-cough, but thinks it likely to prove of more value in cases of children of twelve years and upwards than in those younger, as they will inhale it more promptly and properly. His mode of exhibition is worthy of attention. "In order to avoid the possibility of an over dose, I have never given the chloroform on a handkerchief, or by means of an inhaler, but have directed the mother, (in case of young children,) or the patient, to spill a little, say about thirty drops, in the palm of the hand, and hold this before the mouth and nose sufficiently near to inhale it fully, but not so close as to exclude a portion of atmospheric air. The best time to begin is just as the patient feels the irritation in the chest increasing to a cough, but if possible, before the cough commences; and the inhalation should be repeated with each return of irritation, unless headache be produced."—*From the N. H. Journal of Medicine.*

